# The efficacy of chlorhexidine gel in the prevention of alveolar osteitis after mandibular third molar extraction: a systematic review and meta-analysis

**DOI:** 10.1186/s12903-017-0376-3

**Published:** 2017-05-19

**Authors:** Amare Teshome

**Affiliations:** Department of Dentistry, School of Medicine, College of Medicine and Health Sciences, University of Gondar, Gondar, P.O.BOX: 196, Ethiopia

**Keywords:** Alveolar osteitis, Dry socket, Chlorhexidine gel, Intervention group, Control group, Systematic review/meta-analysis

## Abstract

**Background:**

Alveolar osteitis is a very painful and distressing condition for a patient who has recently undergone a tooth extraction and has led dental professionals to search for preventive measures. The aim of this meta-analysis to determine the effect of chlorhexidine (CHX) gel on the incidence of alveolar osteitis after mandibular third molar extraction.

**Methods:**

Studies were searched for on electronic search engines using Medline (PubMed), Cochrane central, Scopus and advanced Google Scholar from May 2015 to December 2015. Randomized controlled trial studies with a history of mandibular third molar extraction, along with the administration of topical chlorhexidine gel were included. The risk of bias of the selected articles was assessed using the Cochrane risk of bias assessment tool. RevMan 5.3 Software was used to analyze the pooled effect. I^2^ was calculated to determine heterogeneity and a funnel plot was used to check the risk of bias. Subgroup analysis was also done based on the presence of confounding factors (smoking, oral contraceptive etc.) and on split mouth design.

**Results:**

Out of 52 articles, ten met the inclusion criteria. 862 participants were involved in the selected studies with a mean age range from 24.15 ± 5.02 to 36.65 ± 11. The overall RR was 0.43 (95% CI: 0.32, 0.58, *p* < 0.00001). Three studies used a split-mouth design to check the effect of chlorhexidine gel in the prevention of alveolar osteitis incidence. There was a pooled effect of 0.29 (95% CI: 0.16, 0.50) for the intervention group in the split mouth design studies. A stratified analysis was done to check the effect of CHX gel in patients with confounding factors and a significant reduction of AO incidence was found; 0.60 (95% CI: 0.41, 0.87; *p* = 0.05) in the intervention. There was no reported adverse reaction. The heterogeneity (I^2^) was 40%. The funnel plot showed that there was no significant publication bias.

**Conclusion:**

This meta-analysis suggests that CHX gel is superior to a placebo in reducing the incidence of alveolar osteitis after mandibular third molar extraction.

## Background

Alveolar osteitis (AO) is a poorly understood form of post-operative pain located in or around the area of extracted tooth, which occurs due to a partial or total loss of a blood clot, between the first and third postoperative days [1]. The incidence of AO was reported to be 3–4% and its value may be extended to 45% during the extraction of an impacted tooth [1]. AO more commonly occurs in the mandible, in women (5:1) [2] and for posterior tooth extraction [3].

Mandibular impacted tooth extraction is a routine task carried out by dental surgeons. The procedure causes associated postoperative complications; edema, pain, trismus and AO [4]. Even if the etiology of AO is debated, it may be multifactorial [5]. Some precipitating factors were recognized; hypovascularity due to the density of the bone, anesthetic agents (vasoconstriction), systemic conditions/disease, smoking, age, oral contraceptive (OCP) and traumatic extraction [2, 6, 7]. It occurs due to an increased local fibrinolysis which leads to disintegration of the clot and characterized by severe pain [8].

AO is a self-limiting condition but requires several follow-up visits to the dental clinic due to its severe pain and increases patient’s morbidity and cost of treatment. The treatment goal of AO includes reduction of pain until the socket is healed, prevent bacterial growth and control bleeding. Treatment choices for AO are limited, but the use of eugenol dressing, chlorhexidine (CHX), antibiotics, analgesics, lidocaine gel irrigation of the socket are few of the methods to reduce the incidence of AO [9–11].

Due to its nature of severe pain, prevention of AO decreases the morbidity and cost of treatment and reduce patients repeated dental visit. Different modalities have been investigated in an attempt to prevent the incidence. However, a great controversy still exists regarding the most appropriate and effective method [1, 2]. Some literature examined the effect of CHX gel on AO prevention due to its broad spectrum activity and covers anaerobes and there was no registered resistance [12]. However, some literature showed it’s ineffective in preventing AO occurrence [13, 14]. Due to this controversy, there is no single method which gets universal acceptance and success in the attempt to reduce AO [13].

This systematic review and meta-analysis focused on summarizing literature done on the efficacy of CHX gel in the prevention of AO incidence after mandibular third molar extraction, to carry subgroup analysis of the efficacy of CHX gel on AO in patients with possible confounding factors. This meta-analysis was designed to test the null hypothesis that CHX gel is not effective in the prevention of AO.

### Picos

Is CHX gel postoperatively, (compared with no CHX gel) prevent the incidence of AO after mandibular third molar extraction?

## Methods

### Protocols and registration

The systematic review was done using the preferred reporting items for systematic reviews and meta-analysis (PRISMA) checklist [15]. There was no registration done either for the protocol or the systemic review.

### Literature search

Three databases (Medline/PubMed, Cochrane central, and Scopus) and advanced Google scholar were searched for studies published between January 2010 and December 2015 using the following key terms; “alveolar osteitis”, “CHX gel”, “Dry socket” CHX gel and alveolar osteitis to identify RCTs investigating the effect of CHX gel on AO incidence.

### Eligibility criteria

The included studies were Randomized controlled trial studies (RCTs) which studied the effectiveness of CHX gel for the prevention of AO incidence in mandibular third molar extraction. Only studies that were full-text articles and published in English (as translation funding was not available) were included in the systematic review and meta-analysis. Only published papers were considered for the inclusion. The outcome of the included studies should be AO. Excluded studies were; Studies that deal on AO incidence other than mandibular 3^rd^ molars; papers published before 2010, unpublished papers and non-full text articles.

### Study selection

In this systematic review and meta-analysis RCTs published on intervention studies reporting the effect of CHX gel on AO incidence and publish on English were included. To identify relevant articles; titles and abstracts of retrieved papers were exported to Endnote where duplicates were identified and removed by one reviewer (AT).

**Fig. 1:**
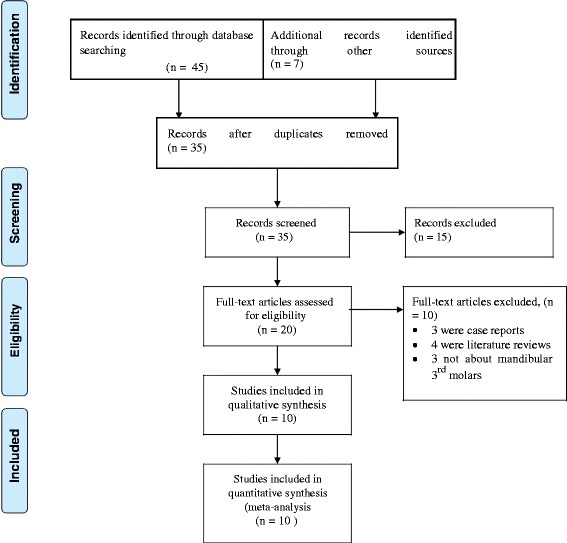
Flow diagram showing the article selection process

All retrieved articles were evaluated by two reviewers independently. A conflict between the reviewers was resolved by consensus. After excluding the non-eligible articles, they were screened according to their titles and abstracts. Full-text articles were assessed and the articles which didn’t meet the inclusion criteria were excluded (Fig. 1).

### Type of intervention

The intervention group receives CHX gel in the socket after extraction once while the control group receives a placebo gel in the extracted socket immediately.

### Risk of bias assessment

**Fig. 2:**
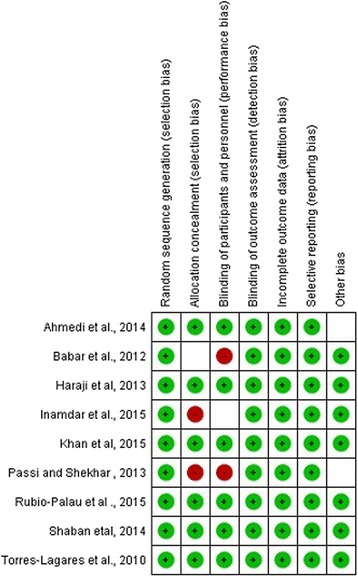
Risk of bias assessment for RCTs

The risk of bias was assessed based on the Cochrane collaboration’s tool for assessing the risk of bias for RCTs [16]. The status of bias of each included trial was assessed both at the study and outcome level with the following parameters; random sequence generation, allocation concealment, blinding of the examiner and/or patient, incomplete data outcome and loss to follow-up (Fig. 2).

### Data extraction and analysis

Data was extracted from the selected 10 articles using a format prepared in Microsoft Excel Spreadsheet and transferred to RevMan 5.3 [17] for meta-analysis. Data extracted from each RCTs on [1] Demographic characteristics (including mean age, sample size, country of the study done); [2] diagnosis of AO, [3] type of intervention and [4] the type of outcomes (Table 1). Heterogeneity between trials was assessed using the I^2^ statistic. Heterogeneity was considered substantial if I^2^ was greater than 50% and a random effects model applied; otherwise, a fixed effects model was used for the analysis. The pooled data for each outcome were reported as Risk ratio (RR). The publication bias of the articles was assessed using funnel plot.

**Table 1:** Study characteristics of the included studies in the systemic review/meta-analysis

Authors/years	Country	Sample size	Type of study	Mean age	Diagnosis of alveolar osteitis	Interventions		Outcomes		Results
						Experimental groups	Control group	Experimental	Control	
Torres-Lagares et al., 2010 [18]	Spain	expermental:24 Control: 14	Randomized, double-blinded study	Average age 32 (ranged, 18 to 57)	Blum’s criteria	0.2% Chlorehixidine (CHX) gel	Gel placebo	1 case of alveolar osteitis	4 cases of alveolar osteitis	The difference was not statistically Significant (*p* = 0.402).
Babar et al., 2012 [24]	Pakistan	Experimenta: 50 Control:50	Randomized controlled trials	29 (+6 age) (Range 18 to 40 years)	Blum’s criteria	0.2% CHX gel + (ibuprofen 400 mg)	No treatment + ibuprofen 400 mg)	4 cases with alveolar osteitis	14 cases with alveolar osteitis	A single application of CHX gel was effective in reducing frequency of alveolar osteitis following mandibular third molar surgery.
Khan et al., 2015 [29]	USA	Experimental: 128 Contro:125	Double-blinded Randomized clinical trial	36.65 (±11 year)	Blum’s criteria Visual Analog Scale (VAS),	0.2%CHX gel	Placebo gel	7 cases with alveolar osteitis	23 cases with alveolar osteitis	Therefore, application of CHX in generalized Is recommended.
Freudenthal, 2015 [23]	Sweden	expermental48 Control:47	Double-blinded randomized	19 to 65 years: age range 33 years (SD, 10.3 years)	Blum’s criteria	0.2% CHX gel	Placebo gel	11 cases with alveolar osteitis	9 cases with alveolar osteitis	Did not verify that application of CHX gel improves healing
Passi and Shekhar, 2013 [25]	Lucknow, U.P	expermental:40 Control:40	Clinical trial	NA	Blum’s criteria	0.2%CHX gel Amoxacilline/clavulanic acid, Metronidazole	0.9% normal saline	1 case with alveolar osteitis	3 cases with alveolar osteitis	Reduce the incidence of alveolar osteitis after the extraction of impacted mandibular third molars by approximately30-40%.
Shaban et al., 2014 [21]	Iran	expermental: 41 Control:41	Double-blind Clinical Trial	24.15 ± 5.02 Age range : 18–35	Blum’s criteria	0.2% CHX gel	No	2 cases with alveolar osteitis	9 cases with alveolar osteitis	The frequency of alveolar osteitis was significantly lower in sockets receiving the CHX gel in comparison to control sockets (RR = 0.22, 95%CI:0.06–0.71)
Rubio-Palau et al., 2015 [19]	Spain	expermenta:80 Control:80	Double-blind Clinical Trial	25.04 mean age	Blum’s criteria	0.2% CHX gel	Bioadhesive placebo	14 cases with alveolar osteitis	18 cases with alveolar osteitis	Reduced the frequency of AO by 22.22% compared to the control group
Ahmedi et al., 2014 [22]	Kosovo	expermental:25 Control:25	A randomized Split-mouth-design		Blum’s criteria	1% CHX gel	Saline solution	1 case with alveolar osteitis	7 cases with alveolar osteitis	The application of CHX gel 1% may significantly reduce the incidence of DS following third molar extraction.
Inamdar et al., 2015 [26]	India	expermental:20 Control:10	Comparative Randomized Prospective Study		Blum’s Criteria	10 patients receive CHX gel; 10 patients revive ornidazole gel	No treatment	1 case with alveolar osteitis	2 cases with alveolar osteitis	The incidence of avolar osteitis is significantly less on placement of CHX gel
Haraji et al., 2013 [20]	Iran	Expermental : 40 Control:40	Double-blinded split-mouth randomized study	18–45 years (Age range) 21.6 ± 2.5 years	Blum’s standardized criteria	0.2% CHX gel	Placebo Gel	9 cases with alveolar osteitis	26 cases with alveolar osteitis	Single-dose intra-alveolar application of CHX gel can reduce dry socket incidence.

In addition, subgroup analysis was done on patients with possible confounding factors (smoking, Oral contraceptive (OCP), and on split-mouth design studies.

## Results

### Study selection

A total of 52 potential studies were retrieved from the initial literature search using three electronic databases (Medline/PubMed, Cochrane central and Scopus) and Advanced Google scholar. After removal of duplicates, 35 articles remained. Out of 35, fifteen articles were excluded during the title and abstract screen and the remaining 20 full-text Articles were selected and assessed in more detail for eligibility. Finally, ten articles met the inclusion criteria and were included in the systematic review and Meta-analysis (Fig. 1).

### Study characteristics

#### Methods

All ten studies selected for the systematic review and Meta- analysis were RCTs published in English between January 2010 and December 2015. Two of them were conducted in Spain, two studies in Iran and one each in Pakistan, USA, Sweden, U.P, Kosovo, and India. All of the studies included patients having a mandibular third molar extraction (Table 1).

#### Participants

All 10 trials involved a total of 862 participants and investigated the efficacy of the CHX gel on the reduction of AO incidence after mandibular third molar extraction. Three studies used split-mouth principle, where there was extraction of mandibular third molar bilaterally and one side serves as a control group whiles the other side serves as the intervention group. The mean age of the participants was ranged from 24.15 (±5.02) to 36.65 (±11) years.

Majority of the studies used Blum’s criteria for diagnosing AO (Table 1). Blum gives a standardized definition for AO which is universally accepted. AO is considered if the post-operative pain is in or around the extraction socket, the severity increases at any time between the 1 and 3 postoperative days, partial or totally disintegrated blood clot within the alveolar socket with or without halitosis.

#### Intervention

All patients in the intervention group received CHX gel (0.2% or 1%) while the control group received a placebo gel (Table 1).

#### Outcomes

In all studies, the primary outcome assessed was the incidence of AO.

#### Risk of bias assessment

The risk of bias within the studies was done using a Cochrane Collaboration’s tool for assessing the risk of bias for RCTs [16]. All of the studies were assessed in terms of random sequence generation, allocation concealment, blinding of participants and outcomes and selective reporting. All of the included studies revealed a randomized sequence of generation and the majority of the studies have concealed allocation (Fig. 2).

**Fig. 3:**
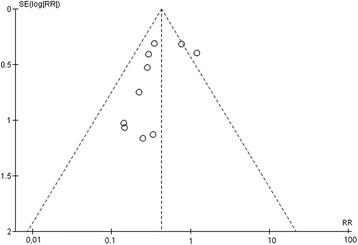
Funnel plot of the included studies

A funnel plot was used to assess the presence of publication bias between the included studies and illustrated a symmetrical spread of the studies with regards to the standard error. This symmetrical distribution showed the presence of low publication bias and also shows high reliability (Fig. 3).

#### Results of individual studies

The efficacy of the CHX gel in the prevention of AO incidence was analyzed from the available RCTs. The majority of the studies (80%) showed a significant reduction of AO incidence in the intervention group after mandibular third molar extraction.

Six studies tried to control the confounding factors like smoking and oral contraceptives while the remaining four studies included smokers/oral contraceptive users in their study and assessed the effect of CHX gel on AO incidence among smokers and/OCP (Table 1).

#### Qualitative analysis of the effectiveness of CHX gels on reduction of AO incidence

A double-blinded RCTs study done in Spain by Torres-Lagares found that there was a reduction of AO incidence in patients received CHX gel but the difference was not statistically significant [18]. However, a study done by Rubio-Palau showed there was a significant reduction of AO incidence in the intervention group (22.22% times compared with the control group) [19].

Three studies (two from Iran [20, 21] and one from Kosovo [22]) tried to assess the efficacy of CHX gel in the prevention of AO after mandibular third molar extraction. There was a significant reduction of AO incidence in a socket with CHX gel compared with the socket with placebo gel.

Other comparative RCTs done in Sweden showed that application of CHX gel has no verified effect on the healing process of AO [23]. Other three RCT studies showed that the application of CHX gel after extraction of mandibular third molar reduces the incidence of AO [24–26] (see Table 1). In the included studies there was no registered adverse.

#### Pooled effect of CHX gels on AO incidence

**Fig. 4:**
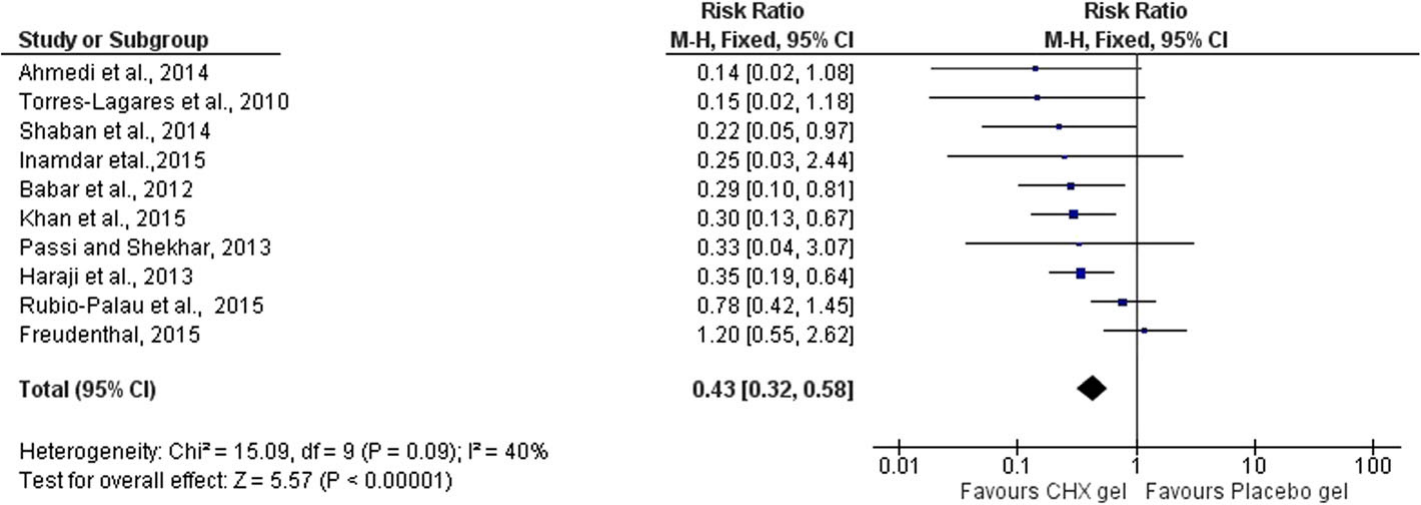
Meta-analysis showing the pooled effect of CHX gel on incidence of AO after mandibular 3^rd^ molar extraction

In this study, the pooled analysis showed a statistically significant reduction of AO in patients taking CHX gel after extraction of a mandibular third molar. Application of CHX gel in the extraction socket after removal of mandibular 3^rd^ molar prevented 57% of AO (RR = 0.43 (95%CI: 0.32, 0.58; *p* < 0.00001) (Fig. 4).

**Fig. 5:**
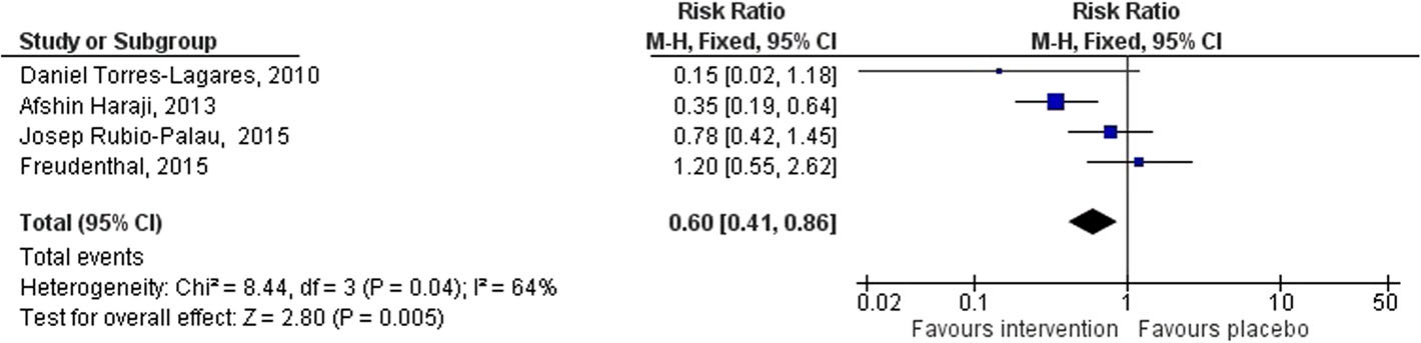
Subgroup meta-analysis showing the effect of CHX gel on AO incidence in patients with confounding factors

Subgroup analysis was done to assess the effect of CHX gel on the prevention of AO incidence in patients with possible confounding factors (smoking and/OCP use) and the pooled effect showed that CHX gel prevented 40% of AO incidence; RR 0.60 (95%CI: 0.41, 0.87; *p* = 0.007) (Fig. 5).

**Fig. 6:**
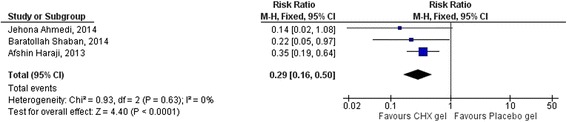
Subgroup meta-analysis showing the effect of CHX gel in AO incidence in split-mouth design studies

Out of the selected RCTs, three studies had a split-mouth design and showed CHX prevented 71% of AO incidence in the intervention; RR = 0.29 (95%CI: 0.16, 0.50;*P* < 0.0001) (Fig. 6).

##### Analysis of the heterogeneity

The I^2^ was 40%, which was not substantial and fixed effect model was considered for the analysis (*p* < 0.00001). The funnel plot was found to be symmetrical and showed there was no significant publication bias.

## Discussion

This meta-analysis includes 10 RCTs that assess the efficacy of CHX gel to prevent the incidence of alveolar osteitis after mandibular third molar extraction. The control group receives only placebo gel.

Previous studies found the incidence of dry socket was highest in the third and fourth decades of life [27], which is in agreement with the present study where the mean age range of the patients was 24.15 ± 5.02 to 36.65 ± 11. This might be due to the presence of well-developed alveolar bone and the relative infrequency of periodontal diseases in this age group makes tooth extraction more difficult.

A meta-analysis done by Ren and Malmsrom showed that antibiotics reduce the incidence of alveolar osteitis when the first dose was given before surgery [28]. Although antibiotics may decrease the incidence of dry socket, antibiotics should not be used in preventing or treating dry socket in a non-immune-compromised subject, due to the potential for development of resistant strains to the antibiotics and other side effects such as hypersensitivity [29, 30]. In this meta-analysis one study used amoxicillin/clavulanic acid and metronidazole in addition to CHX gel while one study used ornidazole gel in the intervention group.

A meta-analysis done by kalantar Motamedi and Khazaei [31], included 7 clinical trials with 593 participants and found Bioadhesive 0.2% CHX gel prevented approximately 72% of AO (OR = 0.28,95%CI:0.18–0.44; *p* < 0.001). This is relatively comparable with the results of the present study, in which the administration of CHX gel in the extraction socket after mandibular 3^rd^ molar extraction prevents 57% of AO incidence (RR = 0.43, 95%CI:0.32–0.58, *p* < 0.00001). also, Hedstrom and Sjogren [32] found similar result with the present study, which showed 0.12% chlorhexidine mouth rinse twice daily after mandibular 3^rd^ molar extraction reduced the incidence of AO.

Some predisposing factors increase the incidence of AO after mandibular 3^rd^ molar extraction such as density of the bone, anesthetic agent, systemic disease, smoking, age, OCP and traumatic extraction [2, 6, 7]. In the present study 3 studies were with confounding factors (smoking and oral contraceptive use. This factors increase the incidence of AO after mandibular 3^rd^ molar extraction due to increase in estrogen in OCP and the chemicals in cigarettes, which can prevent your body from healing. This study tried to do stratified analysis by the presence of possible confounding factors (smoking, OCP, etc.). This stratified analysis has found a statistically significant reduction of AO in patients with confounding factors, RR was 0.60 (95%CI: 0.41-0.87; *p* = 0.007). This revealed CHX gel has an efficacy of preventing AO in patients with confounding factors after extraction of a mandibular 3^rd^ molar.

Out of the selected studies, three studies used spit-mouth design. Both the intervention and controlled groups were in the same patient (the left and right jaw) and the possible confounding factors might be controlled. The pooled effect showed that CHX gel prevented 71% of AO (RR = 0.29, 95%CI: 0.16–0.50) in the socket that received CHX gel compared with the socket received placebo gel. There was no heterogeneity between the studies (I^2^ = 0).

This systemic review and meta-analysis faced some limitations; first article search was done only on papers published in the English language due to the financial problem for interpretation and this may lead bias of selection. The second limitation was most of the studies have a lack of data regarding the surgery difficult index of the tooth. Due to this gap, it was difficult to do a subgroup analysis based on the difficulty index and show the effect of it on AO incidence.

## Conclusion

This systematic review and meta-analysis provide clinically significant evidence that CHX gel application in the extraction socket of mandibular 3^rd^ molar has reduced the incidence of AO.

## Abbreviations

AO: Alveolar osteitis
CHX: Chlorhexidine
CI: Confidence interval
RCTs: Randomized controlled trial studies
RR: Risk ratio

## References

[1] Blum IR (2002). Contemporary views on dry socket (alveolar osteitis): a clinical appraisal of standardization, aetiopathogenesis and management: a critical review. Int J Oral Maxillofac Surg.

[2] Kolokythas A, Olech E, Miloro M (2010). Alveolar osteitis: a comprehensive review of concepts and controversies. Int J Dent.

[3] Jaafar N, Nor GM (2000). The prevalence of post-extraction complications in an outpatient dental clinic in Kuala Lumpur Malaysia--a retrospective survey. Singapore Dent J.

[4] Susarla SM, Blaeser BF, Magalnick D (2003). Third molar surgery and associated complications. Oral Maxillofac Surg Clin N Am.

[5] MacGregor AJ (1968). Aetiology of dry socket: a clinical investigation. Br J Oral Surg.

[6] Bonine FL, Larsen PE (1995). Effect of chlorhexidine rinse on the incidence of dry socket in impacted mandibular third molar extraction sites. Oral Surg Oral Med Oral Pathol Oral Radiol Endodontology.

[7] Abu Younis MH, Abu Hantash RO. Dry socket: frequency, clinical picture, and risk factors in a palestinian dental teaching center. Open Dent J. 2011;5(1):7–12. 10.2174/1874210601105010007 PMC3089956 21559187

[8] Daly B, Sharif MO, Newton T, Jones K, Worthington HV. Local interventions for the management of alveolar osteitis (dry socket). Cochrane Database Syst Rev. 2012;(12). Art. No.:CD006968. doi:10.1002/14651858.CD006968.pub2. 10.1002/14651858.CD006968.pub2 23235637

[9] Torres-Lagares D, Serrera-Figallo MA, Romero-Ruiz MM, Infante-Cossío P, García-Calderón M, Gutiérrez-Pérez JL (2004). Update on dry socket: a review of the literature. Med Oral Patol Oral Cirugia Bucal.

[10] Lagares DT, Cossio PI, Perez JLG, Ruiz MMR, Garcia M (2006). Intra-alveolar chlorhexidine gel for the prevention of dry socket in mandibular third molar surgery. A pilot study Med Oral.

[11] Karnure MH (2013). Review on conventional and novel techniques for treatment of alveolar osteitis. Asian J Pharm Clin Res.

[12] Lang N, Brecx MC (1986). Chlorhexidine digluconate–an agent for chemical plaque control and prevention of gingival inflammation. J Periodontal Res.

[13] Caso A, Hung L-K, Beirne OR (2005). Prevention of alveolar osteitis with chlorhexidine: a meta-analytic review. Oral Surg Oral Med Oral Pathol Oral Radiol Endodontology.

[14] Yengopal V, Mickenautsch S (2012). Chlorhexidine for the prevention of alveolar osteitis. Int J Oral Maxillofac Surg.

[15] Moher D, Liberati A, Tetzlaff J, Altman DG (2009). Preferred reporting items for systematic reviews and meta-analyses: the PRISMA statement. Ann Intern Med.

[16] Higgins JP, Altman DG, Gøtzsche PC, Juni P, Moher D, Oxman AD, et al. The Cochrane Collaboration’s tool for assessing risk of bias in randomised trials. BMJ. 2011;343:1–9;d5928. 10.1136/bmj.d5928 PMC3196245 22008217

[17] Review Manager (RevMan) [Computer program]. Version 5.3. Copenhagen: The Nordic Cochrane Centre, The Cochrane Collaboration; 2014.

[18] Torres-Lagares D, Gutierrez-Perez JL, Hita-Iglesias P, Magallanes-Abad N, Flores-Ruiz R, Basallote-Garcia M (2010). Randomized, double-blind study of effectiveness of intra-alveolar application of chlorhexidine gel in reducing incidence of alveolar osteitis and bleeding complications in mandibular third molar surgery in patients with bleeding disorders. J Oral Maxillofac Surg.

[19] Rubio-Palau J, Garcia-Linares J, Hueto-Madrid J-A, González-Lagunas J, Raspall-Martin G, Mareque-Bueno J (2015). Effect of intra-alveolar placement of 0.2% chlorhexidine bioadhesive gel on the incidence of alveolar osteitis following the extraction of mandibular third molars. A double-blind randomized clinical trial. Med Oral Patol Oral Cirugia Buca.

[20] Haraji A, Rakhshan V, Khamverdi N, Alishahi HK (2013). Effects of intra-alveolar placement of 0.2% chlorhexidine bioadhesive gel on dry socket incidence and postsurgical pain: a double-blind split-mouth randomized controlled clinical trial. J Orofac Pain.

[21] Shaban B, Azimi HR, Naderi H, Janani A, Zarrabi MJ, Nejat A (2014). Effect of 0.2% chlorhexidine gel on frequency of dry socket following mandibular third molar surgery: a double-blind clinical trial clinical trial. J Dent Mater Tech.

[22] Ahmedi J, Ahmedi E, Agani Z, Hamiti V, Reçica B, Tmava-Dragusha A. The efficacy of 1% chlorhexidine gel on the reduction of dry socket occurence following surgical third molar extraction—pilot study. Open J Stomatol. 2014;4(3):152–60.

[23] Freudenthal N, Sternudd M, Jansson L, Wannfors K (2015). A double-blind randomized study evaluating the effect of intra-alveolar chlorhexidine gel on alveolar osteitis after removal of mandibular third molars. J Oral Maxillofac Surg [Internet].

[24] Babar A, Ibrahim MW, Baig NJ, Shah I, Amin E (2012). Efficacy of intra-alveolar chlorhexidine gel in reducing frequency of alveolar osteitis in mandibular third molar surgery. J Coll Physicians Surg Pak.

[25] Deepak P, Pratishtha S (2013). Effects of a chlorhexidine gluconate oral rinse on the incidence of alveolar osteitis in mandibular third molar surgery. Eur J Dent Ther Res.

[26] Inamdar MN, Chauhan R, Mapare SA, Goswami RP, Goswami Y, Khadri SF (2015). Prevention of dry socket using chlorhexidine gel and ornidazole gel in impacted mandibular third molar: a comparative randomized prospective study on 30 patients. J Int Oral Health.

[27] Oginni FO, Fatusi OA, Alagbe AO (2003). A clinical evaluation of dry socket in a Nigerian teaching hospital. J Oral Maxillofac Surg Off J Am Assoc Oral Maxillofac Surg.

[28] Ren Y-F, Malmstrom HS (2007). Effectiveness of antibiotic prophylaxis in third molar surgery: a meta-analysis of randomized controlled clinical trials. J Oral Maxillofac Surg Off J Am Assoc Oral Maxillofac Surg.

[29] Blum IR. Contemporary views on dry socket (alveolar osteitis): A clinical appraisal of standardization, aetiopathogenesis and management: A critical review. Int J Oral Maxillofac Surg. 2002;31(3):309–17. 10.1054/ijom.2002.0263 12190139

[30] Laird WR, Stenhouse D, Macfarlane TW (1972). Control of post-operative infection. a comparative evaluation of clindamycin and phenoxymethylpenicillin. Br Dent J.

[31] Kalantar Motamedi MR, Khazaei S. Bioadhesive chlorhexidine gel for reduction of alveolar osteitis incidence: Systematic review and meta-analysis of randomized controlled trials. Dent Hypotheses. 2014;5(4):35–40.

[32] Hedström L, Sjögren P (2007). Effect estimates and methodological quality of randomized controlled trials about prevention of alveolar osteitis following tooth extraction: a systematic review. Oral Surg Oral Med Oral Pathol Oral Radiol Endodontology.

